# METTL3 promotes the progression of osteosarcoma through the N6-methyladenosine modification of MCAM via IGF2BP1

**DOI:** 10.1186/s13062-024-00486-x

**Published:** 2024-06-07

**Authors:** Dongjian Song, Qi Wang, Zechen Yan, Meng Su, Hui Zhang, Longyan Shi, Yingzhong Fan, Qian Zhang, Heying Yang, Da Zhang, Qiuliang Liu

**Affiliations:** 1 Department of Pediatric Surgery, The First Affiliated Hospital of Zhengzhou University, No. 1 Jianshe East Road, Zhengzhou, 450052 China; 2 Institute of Molecular Cancer Surgery, Henan Province Engineering Research Center, Zhengzhou University, Zhengzhou, 450052 China; 3 Department of Urology, The First Affiliated Hospital of Zhengzhou University, Zhengzhou, 450052 China

**Keywords:** OS, YY1, METTL3, IGF2BP1, MCAM

## Abstract

**Background:**

The molecular mechanisms of osteosarcoma (OS) are complex. In this study, we focused on the functions of melanoma cell adhesion molecule (MCAM), methyltransferase 3 (METTL3) and insulin like growth factor 2 mRNA binding protein 1 (IGF2BP1) in OS development.

**Methods:**

qRT-PCR assay and western blot assay were performed to determine mRNA and protein expression of MCAM, METTL3, IGF2BP1 and YY1. MTT assay and colony formation assay were conducted to assess cell proliferation. Cell apoptosis, invasion and migration were evaluated by flow cytometry analysis, transwell assay and wound-healing assay, respectively. Methylated RNA Immunoprecipitation (MeRIP), dual-luciferase reporter, Co-IP, RIP and ChIP assays were performed to analyze the relationships of MCAM, METTL3, IGF2BP1 and YY1. The functions of METTL3 and MCAM in tumor growth were explored through in vivo experiments.

**Results:**

MCAM was upregulated in OS, and MCAM overexpression promoted OS cell growth, invasion and migration and inhibited apoptosis. METTL3 and IGF2BP1 were demonstrated to mediate the m6A methylation of MCAM. Functionally, METTL3 or IGF2BP1 silencing inhibited OS cell progression, while MCAM overexpression ameliorated the effects. Transcription factor YY1 promoted the transcription level of METTL3 and regulated METTL3 expression in OS cells. Additionally, METTL3 deficiency suppressed tumor growth in vivo, while MCAM overexpression abated the effect.

**Conclusion:**

YY1/METTL3/IGF2BP1/MCAM axis aggravated OS development, which might provide novel therapy targets for OS.

**Supplementary Information:**

The online version contains supplementary material available at 10.1186/s13062-024-00486-x.

## Introduction

Osteosarcoma (OS) is an aggressive malignant bone tumor originates from original transformed cells of mesenchymal origin, with a high mortality rate in adolescents and children [1, 2]. Approximately 80% of patients with OS exhibit subclinical pulmonary micrometastases at diagnosis [3]. Studies have shown that the overall 5-year survival rate for patients with localized OS is between 65 and 75%, while the overall 5-year survival rate for patients with recurrent and metastatic tumors is only 20% [4]. Despite advances in OS treatment such as adjuvant chemotherapy and surgical resection, many patients still face with local recurrence or distant metastasis [5, 6]. No precise diagnostic markers or effective therapeutic targets have been identified, hampering efforts to improve clinical outcomes for OS. Therefore, it is critical to investigate innovative strategies to improve clinical outcomes of OS.

Melanoma cell adhesion molecule (MCAM) is a transmembrane glycoprotein that mediates cell adhesion by regulating cell-cell and cell-matrix interactions [7]. MCAM is highly expressed in tumor tissues and is thought to mediate tumor development and prognosis. For instance, Zou et al. declared that ST6GAL1 blocked HCC metastasis by affecting the sialylation of MCAM on the cell surface [8]. Chen et al. claimed that SOX18 transactivated MCAM and CCL7 to aggravate gastric cancer metastasis [9]. In OS, MCAM was demonstrated to play an oncogenic role via circ_0097271/miR-640/MCAM pathway [10]. Moreover, Du et al. declared that MCAM predicted the poor prognosis and aggravated the metastasis in OS [11]. Even though, the potential mechanisms of MCAM in OS are largely unclear.

Methyltransferase 3 (METTL3) encodes the 70 kDa subunit of MT-A and MT-A is part of the N6-adenosine methyltransferase, which is involved in post-transcriptional methylation of internal adenosine residues in eukaryotic mRNA to form N6-methyladenosine (m6A) [12]. A previous study showed that METTL3 aggravated the metastasis of OS by promoting the m6A modification of TRAF6 [13]. METTL3 induced circNRIP1 expression via m6A modification to enhance OS malignancy by sponging miR-199a [14]. Moreover, RM2target database showed that MCAM contained the m6A methylation modification sites of METTL3. Nonetheless, whether METTL3 participates in the progression of OS through MCAM is still not investigated.

Insulin like growth factor 2 mRNA binding protein 1 (IGF2BP1) encodes a member of the IGF2BP family and works by binding mRNAs of specific genes to regulate their translation [15]. It has been documented that the abnormal expression of IGF2BP1 is linked to the development of various types of cancers, such as cervical cancer [16], gastric cancer [17] and lung cancer [18]. IGF2BP1 was related to high tumor grade and metastasis of OS [19]. ENCORI database showed that IGF2BP1 affected MCAM. However, the relation between MCAM and IGF2BP1 in OS is not clear.

In this paper, we focused on the functions and relations of MCAM, METTL3 and IGF2BP1 in OS malignant phenotypes, expecting to discover a novel pathway in OS.

## Materials and methods

### Collection of OS tissues

34 OS patients at the First Affiliated Hospital of Zhengzhou University were invited in this study and they were provided the written informed consents. The tumor tissues and adjacent non-tumor tissues were harvested from the participants. The procedures were authorized by the Ethics Committee of the First Affiliated Hospital of Zhengzhou University.

### Cell culture and transfection

OS cell lines (HOS, MG63 and U2OS) and SV40-transfected osteoblasts (hFOB1.19) were bought from Procell (Wuhan, China). HOS and MG63 cells were cultured in NEAA-contained MEM (Procell). U2OS cells were cultured in McCoy’s 5 A (Procell). hFOB1.19 cells were cultured in DMEM/F12 (Procell) + 0.3 mg/mL G418 (Procell). In addition, all the used mediums were added with 10% FBS (Procell) and 1% penicillin-streptomycin (Procell) and placed in a humidity incubator with 5% CO_2_ at 37 °C.

Short hairpin RNA (shRNA) target MCAM (sh-MCAM), METTL3 (sh-METTL3) or IGF2BP1 (sh-IGF2BP1) and their control (sh-NC); MCAM overexpression vector (MCAM), IGF2BP1 overexpression vector (IGF2BP1), YY1 overexpression vector (YY1) and empty control (pcDNA); small interfering RNA against YY1 (si-YY1) and scramble control si-NC were synthesized by RIBOBIO (Guangzhou, China) and transfected to OS cells according to Lipofectamine 2000 (Invitrogen, Carlsbad, CA, USA).

### qRT-PCR

Total RNA was isolated from tissues and cells with the usage of TRIzol reagent (Beyotime, Shanghai, China) and then reversely transcribed into cDNAs by using PrimeScript™ RT reagent (Takara, Dalian, China). qRT-PCR was performed using SYBR Premix Ex Taq II (Takara) and mRNA expression was calculated based on the method of 2^−ΔΔCt^. GAPDH served as the internal interference. The primers were presented in Table 1.

**Table 1:** Primers sequences used for qPCR

Name		Primers for PCR (5’-3’)
MCAM	Forward	CTCTTCCTGGAGCTGGTCAA
	Reverse	CCCATCTCTTCTGGGAGCTTATCT
YY1	Forward	AAGCTGCACTTTCTTGGGGT
	Reverse	ACCATCTTCAGGCAACCAGG
IGF2BP1	Forward	CTCTCGGAGGGGTTTCGGA
	Reverse	CTCTCGTTGAGGTTGCCGAT
METTL3	Forward	CAGAGGCAGCATTGTCTCCA
	Reverse	ATGGACACAGCATCAGTGGG
GAPDH	Forward	AGAAGGCTGGGGCTCATTTG
	Reverse	AGGGGCCATCCACAGTCTTC

### Western blot

The proteins in tissues and cells were extracted using RIPA buffer (Beyotime) and quantified via BCA protein assay kit (Tiangen, Beijing, China). The proteins were subjected to 10% SDS-PAGE (Solarbio, Beijing, China) and then transferred onto PVDF membranes. The membranes were then blocked for 1 h with skimmed milk, incubated overnight with primary antibodies against GAPDH (ab9485; 1:2000), MCAM (ab75769; 1:1000), CyclinD1 (ab16663; 1:200), MMP9 (ab76003; 1:2000), METTL3 (ab195352; 1:1000), IGF2BP1 (ab290736; 1:1000) or YY1 (ab109228; 1:2000) and probed with HRP-conjugated goat anti-rabbit IgG (ab205718; 1:5000) for 1 h. Protein bands were visualized by ECL reagent (Beyotime). The antibodies were bought from Abcam (Cambridge, MA, USA).

### MTT assay

MTT assay was performed to explore OS cell proliferation according to previous study [20]. MG63 and U2OS cells with several transfections were plated into 96-well plates for 48 h and then 10 µL MTT (Solarbio) was pipetted into the well and cultured for 4 h. After that, the formazans were dissolved by adding into DMSO (Solarbio). The OD value at 570 nm was assessed with a microplate reader.

### Colony formation assay

The transfected MG63 and U2OS cells were seeded in 6-well plates and culture for 14 days. The medium was replaced every 3 days. After the colonies were fixed with 4% paraformaldehyde (Solarbio), colored with 1% crystal violet (Solarbio) and then counted.

### Flow cytometry analysis

The cells were suspended in binding buffer and stained with Annexin V-FITC and PI for 15 min in a dark condition. The apoptotic cells were analyzed with a flow cytometry (BD Biosciences, San Jose, CA, USA). All procedures were based on the Annexin V-FITC/PI Apoptosis Detection Kit (Beyotime).

### Transwell assay

After transfection, the cells were cultured for 48 h and then seeded on the top transwell insert chambers (BD Bioscience) with Matrigel (BD Bioscience) pre-coverage. The cell culture medium was added into the lower chamber. After 24 h. the invaded cells were colored with 1% crystal violet (Solarbio) and then visualized with an inverted microscope.

### Wound-healing assay

To investigate cell migration ability, wound-healing assay was conducted as previously described [21, 22]. In brief, the transfected cells were seeded into 12-well plates and the scratch was created with a pipette tip. After incubation for 24 h, the scratch wounds were captured and cell migration was examined with ImageJ 1.8.0.

### Methylated RNA immunoprecipitation (MeRIP) assay

Magna MeRIP m6A Kit (Amyjet Scientific, Wuhan, China) was used for this assay in accordance with the manufacturers’ instructions. In brief, protein A/G beads were coupled with 3 µg anti-M6A antibodies and incubated overnight at 4 °C. Subsequently, the antibody bound beads were immersed in IP buffer containing RNase and protease inhibitors, promoting antibody binding to the target RNAs. Next, the RNAs bound to the antibody were extracted and MCAM enrichment was detected through qRT-PCR.

### Measurement of m6A modification

The content of m6A in MCAM RNA was determined using EpiQuik m6A RNA methylation quantitative kit (Epigentek Group Inc., Farmingdale, NY, USA). The RNAs in sh-NC or sh-METTL3-transfected MG63 and U2OS cells were added to the assay hole along with the m6A standard, followed by the capture and MCAM antibody solution. By measuring the OD value of each hole at 450 nm, m6A level was calculated using colorimetry and determined using a standard curve.

### Dual-luciferase reporter assay

To detect the relation between METTL3 and MCAM, the wild-type (wt) or mutant (mut) MCAM mRNA containing or lacking the m6A modification sites of METTL1 was cloned into pmirGLO plasmid (Promega, Madison, WI, USA), constructing the luciferase reporter vectors MCAM and MUT-MCAM, respectively. Then sh-NC/sh-METTL3 and MCAM/MUT-MCAM were co-transfected into MG63 and U2OS cells.

To investigate the relation between YY1 and METTL3, the wild-type (wt) or mutant (mut) sequences of METTL3 containing or lacking YY1 promoter binding sites were cloned into pmirGLO plasmid (Promega, Madison, WI, USA), constructing the luciferase reporter vectors WT-METTL3 and MUT-METTL3, respectively. Then pcDNA/YY1/si-NC/si-YY1 and WT-METTL3/MUT-METTL3 were co**-**transfected into MG63 or U2OS cells. After 48 h, the luciferase intensity was examined with Dual-Luciferase Reporter Assay Reagent (Promega).

### Actinomycin D (Act D) treatment

After relevant transfection, MG63 cells and U2OS cells were treated with Act D (Sigma-Aldrich, St. Louis, MO, USA) at 0 h, 3 h, 6 h and 9 h. After that, MCAM mRNA expression was examined.

### Co-immunoprecipitation (Co-IP) assay

The PierceTM Co-IP kit (Thermo SCIENTIFIC, Carlsbad, CA, USA) was utilized for this assay [23]. Shortly, MG63 and U2OS cells were lysed for 5 min using IP lysis buffer and then centrifuged for 5 min at 12,000 ×g. The supernatants were added with G-agarose beads coated with antibody against METTL2 (Abcam) or MCAM (Abcam) and maintained for 6 h. The Co-IP proteins were measured by western blot.

### RIP assay

By using Magna RIP RNA-Binding Protein Immunoprecipitation Kit (Millipore, Billerica, MA, USA), RIP assay was performed. Briefly, sh-NC or sh-METTL3-transfected MG63 and U2OS cells were disrupted with RIP buffer and then the supernatants were cultivated with IGF2BP1 antibody (Abcam) or IgG (Abcam) control which was coated with magnetic beads. Next, the RNAs were isolated from the beads and subjected to qRT-PCR for MCAM enrichment.

### ChIP assay

Based on the protocols of EZ ChIP™ Chromatin Immunoprecipitation Kit (Millipore, Bedford, MA, USA), ChIP assay was done. The cross-linked chromatin DNA was firstly treated with ultrasound into fragments of 200–500 bp, and then immunoprecipitation was performed with YY1 primary antibody (Abcam) and IgG negative control. Immunoprecipitated DNA was examined with qRT-PCR.

### *In vivo* experiment

Murine xenograft models were established by injecting the sh-NC, sh-METTL3, sh + METTL3 + pcDNA or sh-METTL3 + MCAM-transfected MG63 cells into the BALB/c nude mice (Shanghai SLAC Laboratory Animals Co., Ltd., Shanghai, China). 8 days later, the tumor volume was monitored every 3 days until 23th and calculated with the formula: (length×width^2^)/2. At 23th day, the mice euthanized for further experiments. The in vivo experiments were approved by the Ethics Committee of Animal Research of the First Affiliated Hospital of Zhengzhou University.

### Immunohistochemical (IHC) analysis

The tissues were fixed with 4% formalin before paraffin embedding. The sections were routinely dewaxed with transparent reagents, hydrated with gradient ethanol, boiled with 0.1 M sodium citrate as antigen, and preserved with 3% hydrogen peroxide to block endogenous peroxidase. Then the sections were blocked with 5% goat serum for 1 h, treated with primary antibody against Ki67 (Abcam), MMP (Abcam) or MCAM (Abcam) and incubated at 4℃ overnight. The sections were then treated at 37℃ for 1 h with the secondary antibody (Abcam). Sections were stained in 3, 3-diaminobenzidine solution (Sigma-Aldrich) for 3 min. Hematoxylin (Sigma-Aldrich) was used as a nuclear counterstain. Microscopes were used to capture images.

### Statistics analysis

Data were analyzed by GraphPad Prism 7 and exhibited as mean ± SD. All experiments were repeated 3 times. Survival curve was generated by Kaplan-Meier plot and analyzed by log-rank test. Difference analysis was conducted by Student’s *t*-test or one-way ANOVA. Linear correlation was analyzed by Spearman’s correlation coefficient analysis [24, 25].

## Results

### MCAM was upregulated in OS tissues and cells

**Fig. 1:**
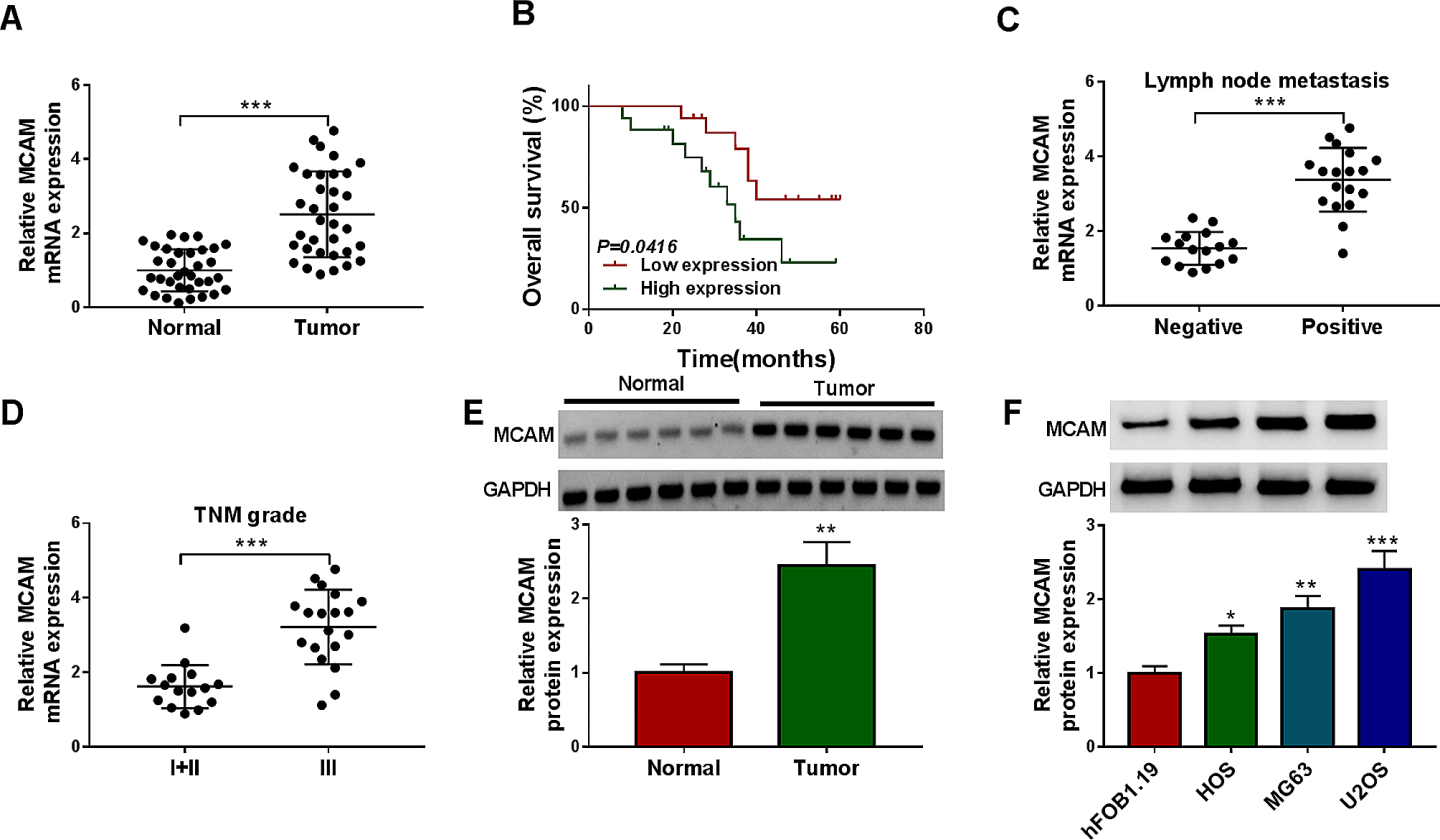
MCAM was overexpressed in OS tissues and cells. (**A**) The mRNA level of MCAM in OS tissues and normal tissues was detected by qRT-PCR. (**B**) The overall survival of OS patients in Low expression group and High expression group was analyzed. (**C**) The expression of MCAM in OS patients with positive or negative lymph node metastasis was determined by qRT-PCR. (**D**) The mRNA expression of MCAM in OS patients in TNM grades III or I+II was determined by qRT-PCR. (**E**) The protein level of MCAM in OS tissues and normal tissues was measured by western blot. (**F**) The protein level of MCAM in hFOB1.19, HOS, MG63 and U2OS cells was measured through western blot. *P < 0.05, **P < 0.01, ***P < 0.001

At the beginning, we conducted qRT-PCR assay to determine the mRNA expression of MCAM in OS tissues (*N* = 34) and normal tissues (*N* = 34). The results indicated that MCAM mRNA level was significantly increased in tumor tissues compared to normal tissues (Fig. 1A). The OS patients were divided into 2 groups according to the expression of MCAM expression, Low expression group and High expression group. It was found that high expression of MCAM was associated with worse prognosis of OS patients (Fig. 1B). Upregulation of MCAM mRNA was observed in lymph node metastasis-positive OS patients compared to lymph node metastasis-negative OS patients (Fig. 1C). The mRNA expression of MCAM in OS patients at TNM grade III was higher expressed than that in OS patients at TNM grades I + II (Fig. 1D). Western blot assay showed that MCAM protein level was increased in OS tissues compared to normal tissues (Fig. 1E). In addition, we determined the protein level of MCAM in OS cells (HOS, MG63 and U2OS) and hFOB1.19 cells by western blot. The results exhibited that MCAM protein level was elevated in HOS, MG63 and U2OS cells compared to hFOB1.19 cells (Fig. 1F). The results indicated that MCAM was abnormally upregulated in OS and related to poor prognosis, lymph node metastasis and advanced TNM grade.

#### MCAM silencing repressed OS cell viability, invasion and migration and promoted apoptosis

**Fig. 2:**
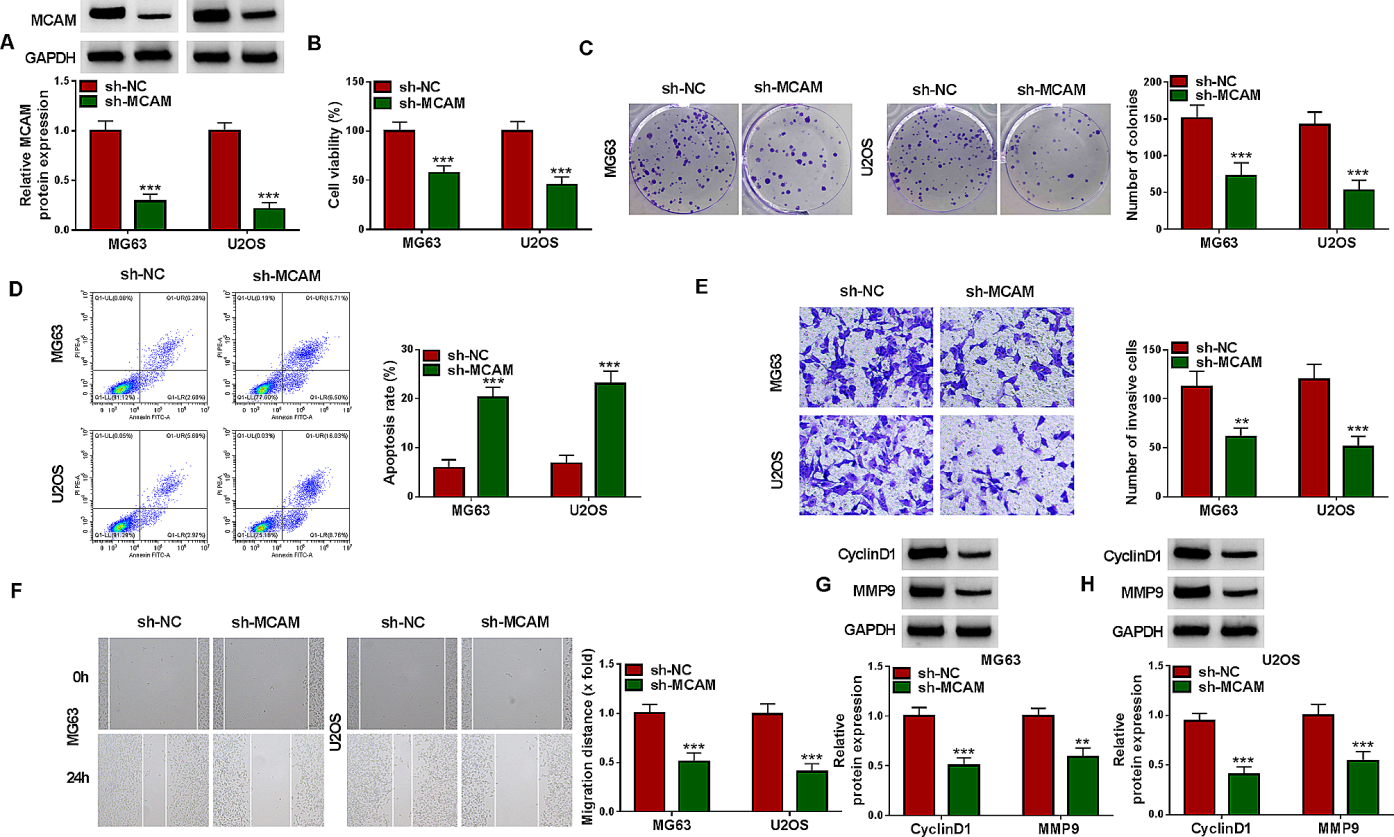
Knockdown of MCAM inhibited cell growth, invasion and migration and facilitated apoptosis in OS cells. MG63 and U2OS cells were transfected with sh-NC or sh-MCAM. (**A**) MCAM protein level in transfected MG63 and U2OS cells was measured by western blot. (**B**) MG63 and U2OS cell viability was evaluated by MTT assay. (**C**) The colony formation ability of MG63 and U2OS cells was assessed by colony formation assay. (**D**) MG63 and U2OS cell apoptosis was analyzed by flow cytometry analysis. (**E**) The invasion of MG63 and U2OS cells was evaluated by transwell assay. (**F**) The migration of MG63 and U2OS cells was examined with wound-healing assay. (**G** and **H**) The protein levels of CyclinD1 and MMP9 in MG63 and U2OS cells were measured via western blot. **P < 0.01, ***P < 0.001

To explore how the aberrant expression of MCAM participate in the progression of OS, MCAM-deficiency MG63 and U2OS cells were constructed by transfecting sh-MCAM into the cells. As shown in Fig. 2A, sh-MCAM transfection led to a significant reduction in MCAM protein level in both MG63 and U2OS cells compared to sh-NC control group, indicating the successful transfection of sh-MCAM. MTT assay indicated that downregulation of MCAM markedly repressed the viability of MG63 and U2OS cells compared to sh-NC control group (Fig. 2B). The colony formation ability of MG63 and U2OS cells was also restrained by silencing MCAM, as indicated by colony formation assay (Fig. 2C). Flow cytometry analysis exhibited that the promotion of cell apoptosis in sh-MCAM-transfected MG63 and U2OS cells (Fig. 2D). As suggested by transwell assay, MCAM knockdown blocked the invasion of MG63 and U2OS cells in comparison with sh-NC control group (Fig. 2E). Wound-healing assay showed that the migration ability of MG63 and U2OS cells was markedly suppressed by MCAM deficiency (Fig. 2F). Moreover, our results showed that MCAM silencing markedly decreased the protein levels of CyclinD1 and MMP9 in MG63 and U2OS cells relative to sh-NC control group (Fig. 2G and H). Collectively, MCAM knockdown suppressed OS cell malignant phenotypes.

### Overexpression of MCAM promoted the proliferation, invasion and migration and inhibited apoptosis of OS cells

**Fig. 3:**
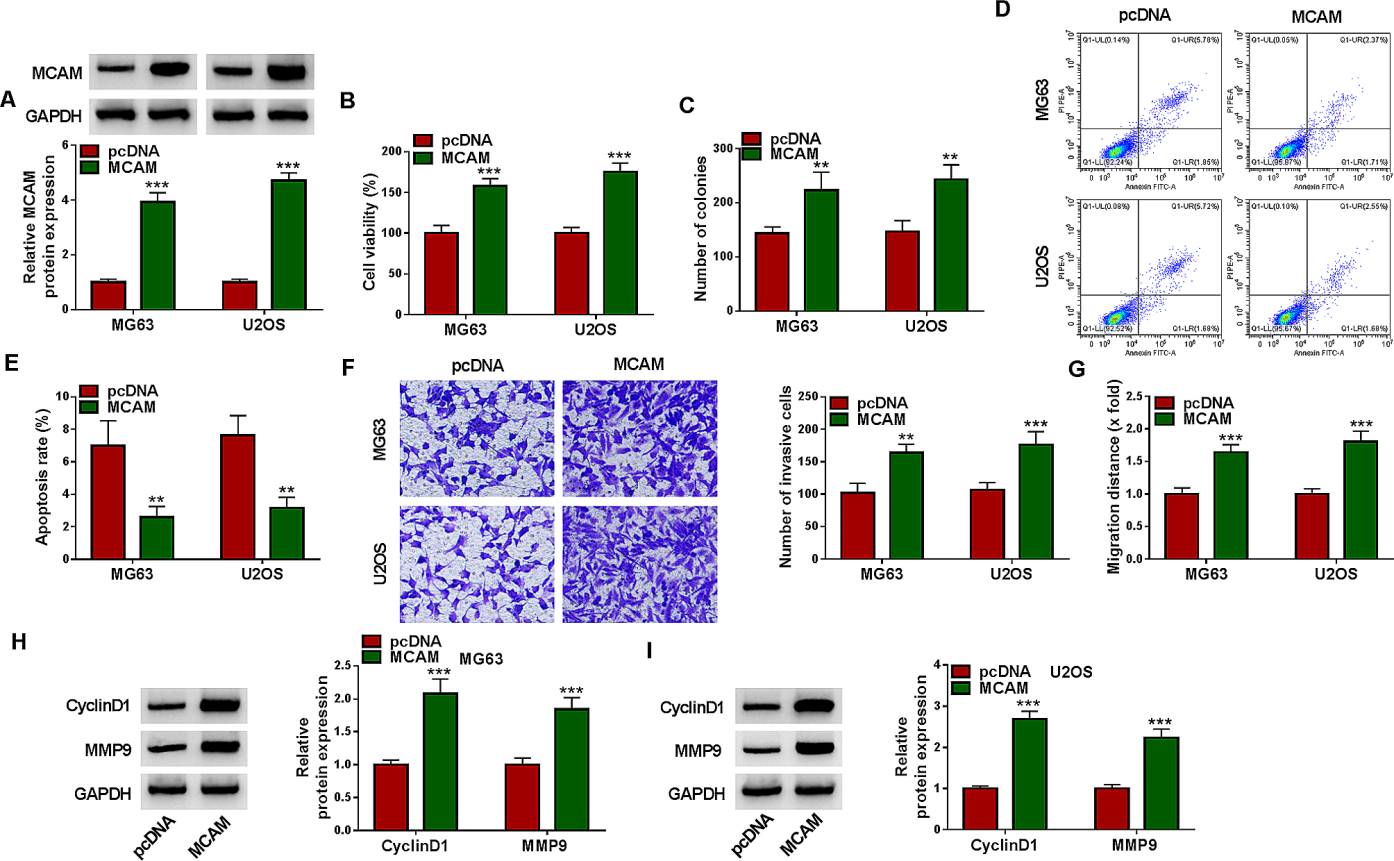
Effects of MCAM overexpression on OS cell proliferation, apoptosis, invasion and migration. MG63 and U2OS cells were transfected with MCAM or pcDNA. (**A**) MCAM protein level in MG63 and U2OS cells was determined via western blot. (**B** and **C**) The viability and colony formation of MG63 and U2OS cells were estimated by MTT assay and colony formation assay. (**D** and **E**) The apoptosis of MG63 and U2OS cells was analyzed by flow cytometry analysis. (**F** and **G**) The invasion and migration abilities of MG63 and U2OS cells were examined by transwell assay and wound-healing assay. (**H** and **I**) The protein levels of CyclinD1 and MMP9 in MG63 and U2OS cells were measured via western blot. **P < 0.01, ***P < 0.001

As presented in Fig. 3A, MCAM overexpression vector tansfection resulted in the marked elevation of MCAM protein level in MG63 and U2OS cells, illustrating the success transfection of MCAM overexpression vector. MTT assay and colony formation assay showed that MCAM upregulation aggravated the viability and colony formation ability of MG63 and U2OS cells compared to pcDNA control group (Fig. 3B and C). Flow cytometry analysis indicated that MG63 and U2OS cell apoptosis was blocked by elevating MCAM expression (Fig. 3D and E). As suggested by transwell assay and wound-healing assay, overexpression of MCAM apparently facilitated the invasion and migration of MG63 and U2OS cells in comparison with pcDNA-transfected cells (Fig. 3F and G). Besides, overexpression of MCAM increased the protein levels of CyclinD1 and MMP9 in both MG63 and U2OS cells relative to pcDNA control cells (Fig. 3H and I). Taken together, MCAM overexpression aggravated OS cell growth and metastasis and repressed apoptosis.

#### METTL3 mediated the m6A methylation modification of MCAM

**Fig. 4:**
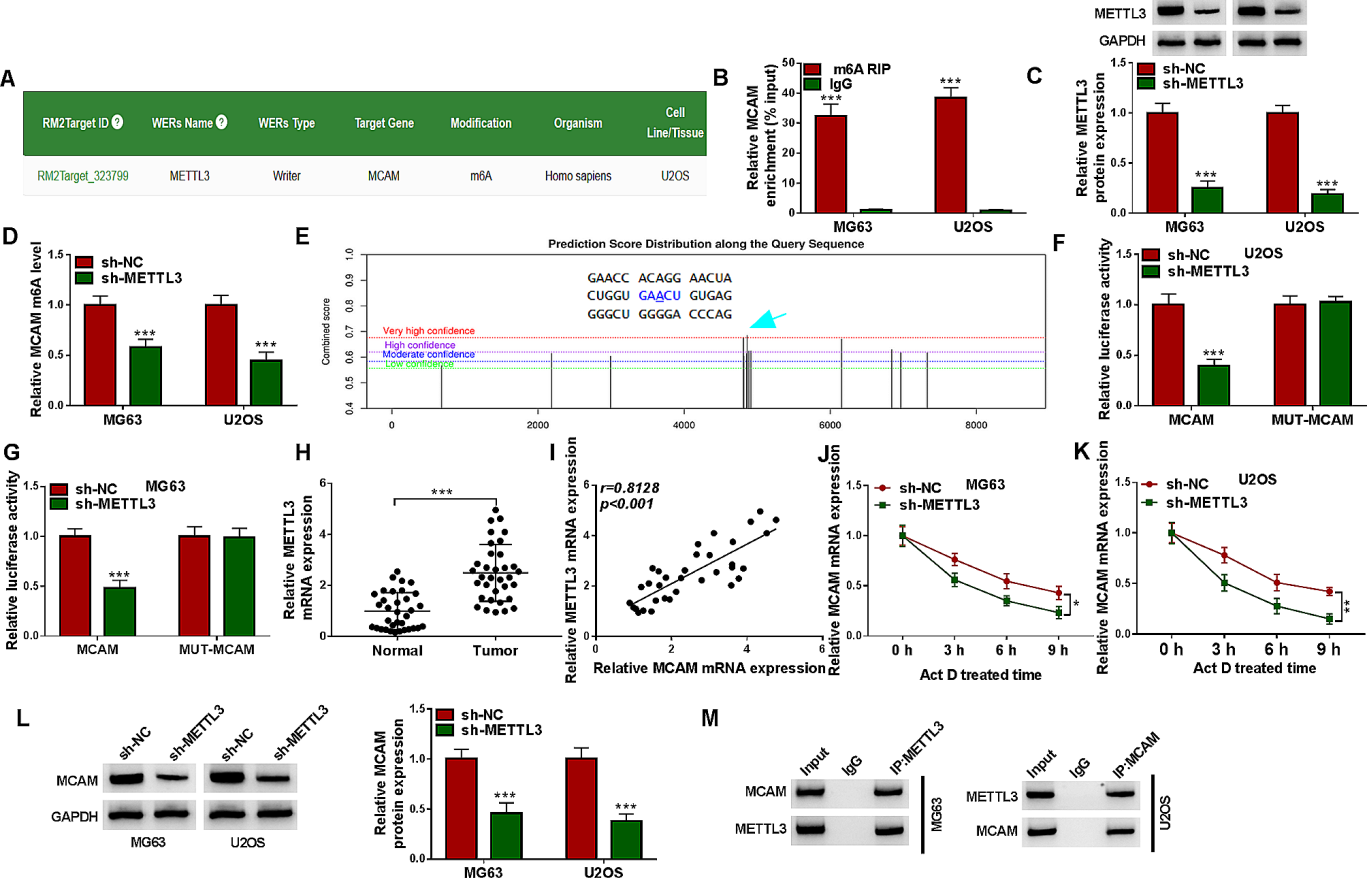
METTL3 targeted MCAM. (**A**) The m6A methylation modification sites of METTL3 in MCAM was predicted by RM2target database. (**B**) After MeRIP, MCAM enrichment was determined by qRT-PCR. (**C**) The protein level of METTL3 in MG63 and U2OS cells transfected with sh-NC or sh-METTL3 was measured via western blot. (**D**) MCAM m6A level in MG63 and U2OS cells transfected with sh-NC or sh-METTL3 was determined. (**E**) SRAMP predicted MCAM mRNA contained the m6A modification site of METTL1. (**F** and **G**) The interaction between MCAM and METTL3 was verified by dual-luciferase reporter assay. (**H**) The mRNA expression of METTL3 in OS tissues and normal tissues was determined by qRT-PCR. (**I**) The linear correlation between METTL3 mRNA level and MCAM mRNA level in OS tissues was estimated by Spearman’s coVrrelation coefficient analysis. (**J** and **K**) After treatment with Act D for 0 h, 3 h, 6 h and 9 h, MCAM mRNA expression was determined by qRT-PCR. (**L**) MCAM protein level in MG63 and U2OS cells with sh-NC or sh-METTL3 transfection was measured via western blot. (**M**) The binding association between MCAM and METTL3 was analyzed by Co-IP assay. *P < 0.05, **P < 0.01, ***P < 0.001

By using RM2target database, MCAM was found to contain the m6A methylation modification sites of METTL3 (Fig. 4A). Through MeRIP assay, we found that MCAM was enriched in m6A methylation (Fig. 4B). As presented in Fig. 4C, sh-METTL3 was transfected into MG63 and U2OS cells, which led to a significant reduction of METTL3 expression. Moreover, our results showed that METTL3 silencing inhibited the level of MCAM m6A in MG63 and U2OS cells (Fig. 4D). Based on SRAMP (http://www.cuilab.cn/sramp/), MCAM mRNA contained the m6A modification site of METTL1 (Fig. 4E). Dual-luciferase reporter assay showed that METTL3 knockdown significantly reduced the luciferase activity of MCAM wild-type luciferase reporter in MG63 and U2OS cells, but the luciferase activity of MUT-MCAM luciferase reporter was not affected (Fig. 4F and G). Subsequently, qRT-PCR was performed to determine the expression of METTL3, showing METTL3 mRNA level was abnormally increased in OS tissues compared to normal tissues (Fig. 4H). As estimated by Spearman’s correlation coefficient analysis, METTL3 mRNA level was positively correlated with MCAM mRNA level in OS tissues (Fig. 4I). Act D treatment showed that METTL3 knockdown could affect the stability of MCAM mRNA in MG63 and U2OS cells (Fig. 4J and K). Moreover, western blot assay showed that METTL3 silencing suppressed the protein level of MCAM in both MG63 and U2OS cells compared to sh-NC control cells (Fig. 4L). Co-IP assay confirmed that METTL3 was also detected in the MCAM immune complex (Fig. 4M). These results indicated that METTL3 regulated MCAM expression via m6A methylation modification.

#### IGF2BP1 mediated M6A methylation modification of MCAM

**Fig. 5:**
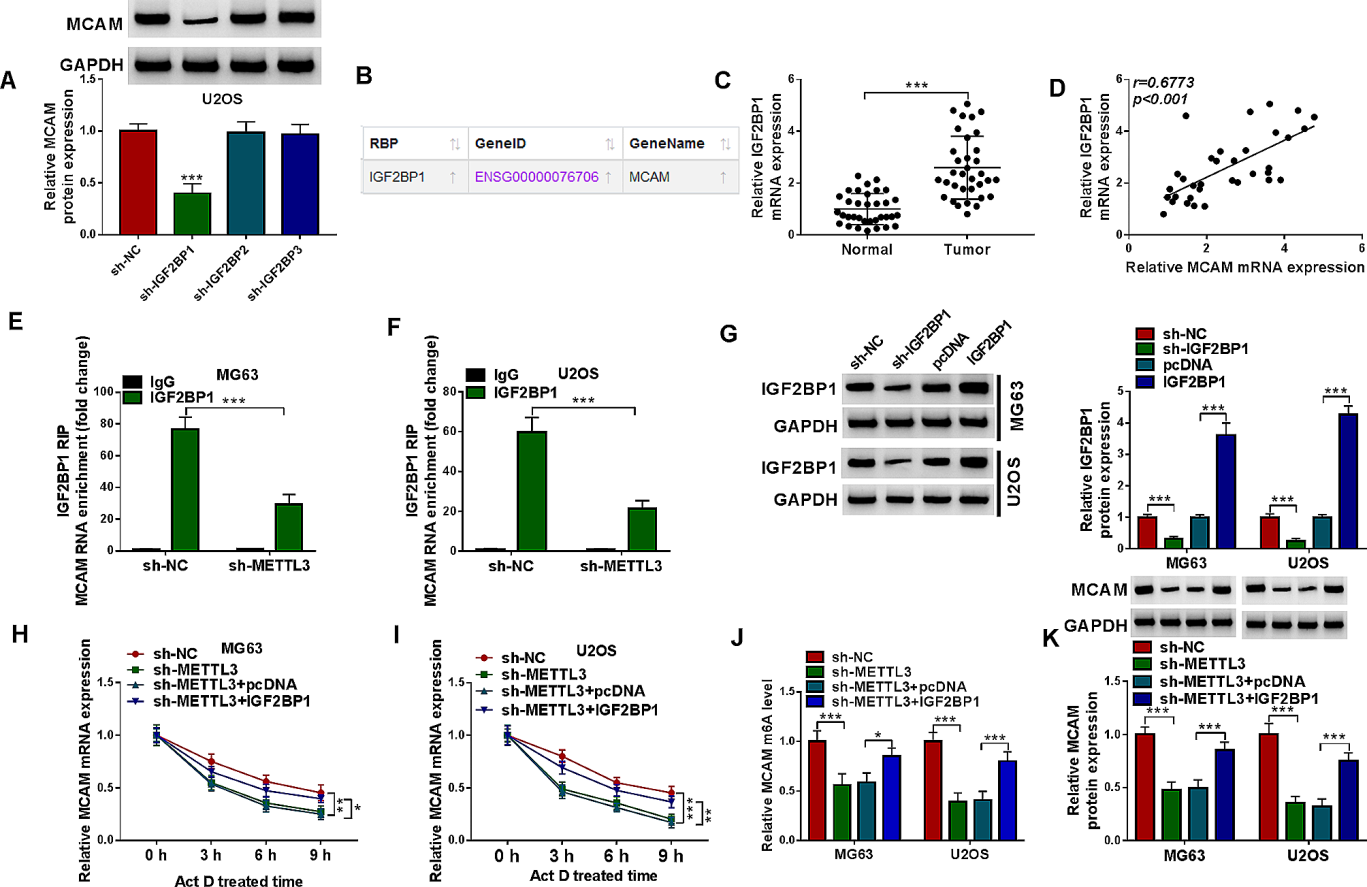
IGF2BP1 affected METTL3-mediated the m6A methylation modification of MCAM. (**A**) After sh-NC, sh-IGF2BP1, sh-IGF2BP2 or sh-IGF2BP3 transfection in MG63 and U2OS cells, MCAM protein level was measured by western blot. (**B**) The relation between IGF2BP1 and MCAM was predicted by ENCORI. (**C**) IGF2BP1 mRNA level in OS tissues and normal tissues was determined by qRT-PCT. (**D**) The linear correlation between the mRNA levels of IGF2BP1 and MCAM in OS tissues was analyzed. (**E** and **F**) The effect of METTL3 knockdown on the interaction between IFG2BP1 and MCAM was estimated by RIP assay. (**G**) The protein level of IGF2BP1 in MG63 and U2OS cells transfected with sh-NC, sh-IGF2BP1, pcDNA or IGF2BP1 was measured by western blot. (**H** and **I**) After Act D treatment, MCAM mRNA level in MG63 and U2OS cells transfected with sh-NC, sh-METTL3, sh-METTL3 + pcDNA or sh-METTL3 + IGF2BP1 was determined by qRT-PCR. (**J**) The effects of METTL3 and IGF2BP1 on MCAM m6A level in MG63 and U2OS cells transfected with sh-NC, sh-METTL3, sh-METTL3 + pcDNA or sh-METTL3 + IGF2BP1 were analyzed. (**K**) MCAM protein level in MG63 and U2OS cells transfected with sh-NC, sh-METTL3, sh-METTL3 + pcDNA or sh-METTL3 + IGF2BP1 was measured by western blot. *P < 0.05, **P < 0.01, ***P < 0.001

As presented in Fig. 5A, IGF2BP1 knockdown suppressed the protein level of MCAM in IGF2BP1 mediates M6A methylation modification of MCAM, whereas IGF2BP2 and IGF2BP3 knockdown had no effect on MCAM protein level. ENCORI database showed that IGF2BP1 affected MCAM (Fig. 5B). Moreover, our results showed that IGF2BP1 mRNA level in OS tissues was upregulated and positively correlated with MCAM mRNA level (Fig. 5C and D). IGF2BP1 RIP assay showed that METTL3 knockdown suppressed IGF2BP1-bound MCAM enrichment in MG63 and U2OS cells, indicating that METTL3 affected the combination between IGF2BP1 and MCAM (Fig. 5E and F). sh-IGF2BP1 or IGF2BP1 overexpression vector was transfected into MG63 and U2OS cells to reduce or elevate IGF2BP1 expression, and the transfection effect was determined by western blot (Fig. 5G). After Act D treatment, sh-METTL3 transfection reduced MCAM mRNA expression in MG63 and U2OS cells, while IGF2BP1 overexpression vector transfection restored the effect (Fig. 5H and I). As presented in Fig. 5J, METTL3 knockdown inhibited MCAM m6A level in MG63 and U2OS cells, while IGF2BP1 elevation rescued the effect (Fig. 5J). Moreover, western blot assay showed that METTL3 silencing decreased MCAM protein level in MG63 and U2OS cells, with IGF2BP1 overexpression rescued the effect (Fig. 5K). Collectively, IGF2BP1 mediated the expression of MCAM affected by METTL3 in OS cells.

#### METTL3 knockdown suppressed OS cell proliferation, invasion and migration and induced apoptosis by modulating MCAM

**Fig. 6:**
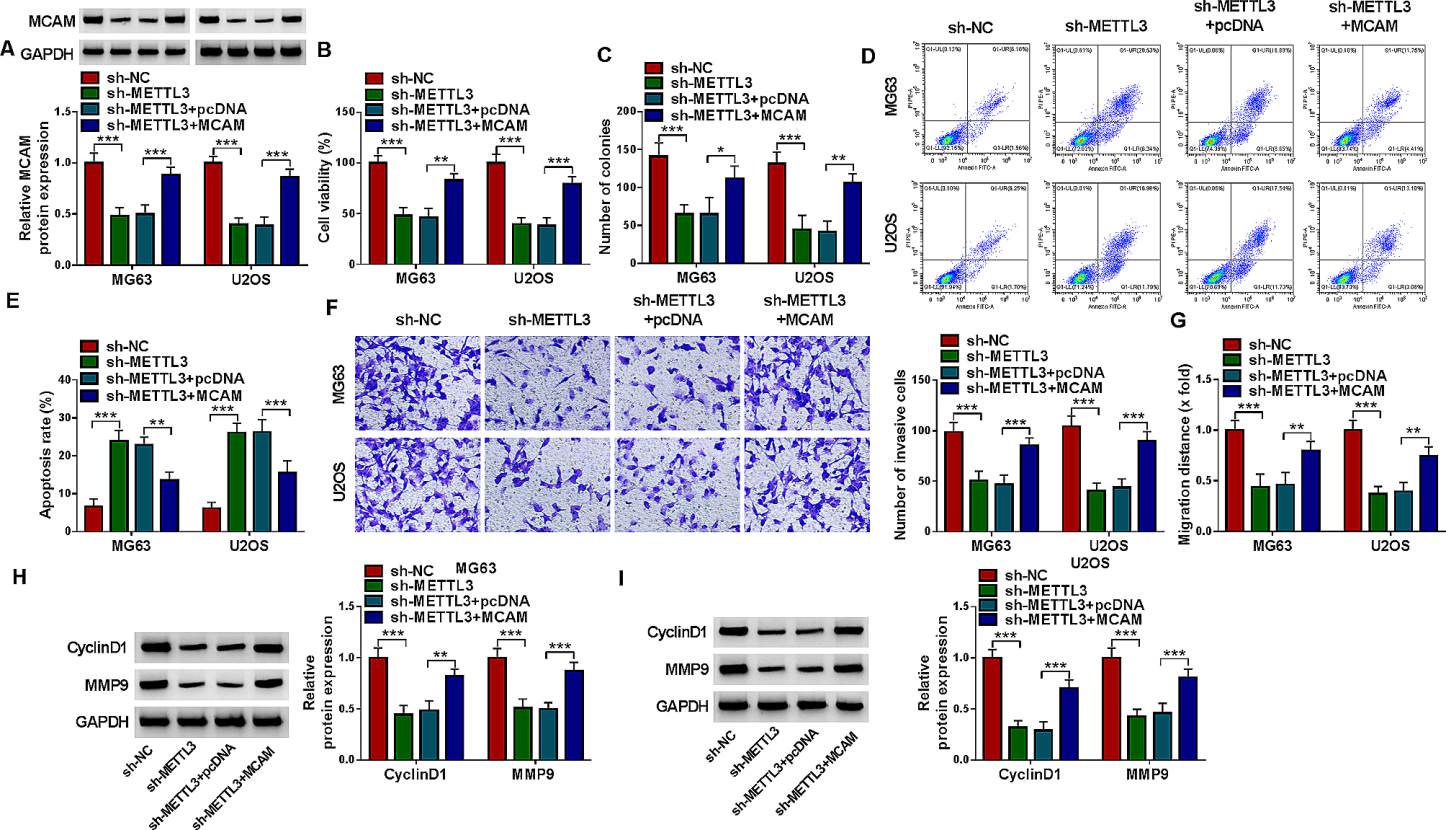
METTL3 deficiency inhibited OS cell progression by altering MCAM expression. MG63 and U2OS cells were transfected with sh-NC, sh-METTL3, sh-METTL3 + pcDNA or sh-METTL3 + MCAM. (**A**) MCAM protein level in MG63 and U2OS cells was measured via western blot. (**B**) MG63 and U2OS cell viability was assessed by MTT assay. (**C**) The colony formation of MG63 and U2OS cells was estimated by colony formation assay. (**D** and **E**) The apoptosis of MG63 and U2OS cells was analyzed by flow cytometry analysis. (**F** and **G**) The invasion and migration of MG63 and U2OS cells were evaluated by transwell assay and wound-healing assay, respectively. (**H** and **I**) The protein levels of CyclinD1 and MMP9 in MG63 and U2OS cells were detected by western blot. *P < 0.05, **P < 0.01, ***P < 0.001

The association between METTL3 and MCAM in OS development was investigated. METTL3 silencing decreased MCAM protein level in MG63 and U2OS cells, while the effect was abolished by transfecting MCAM overexpression vector (Fig. 6A). MTT assay indicated that METTL3 silencing restrained MG63 and U2OS cell viability, but MCAM overexpression restored the effect (Fig. 6B). As illustrated by colony formation assay, MG63 and U2OS cell colony formation ability was repressed by silencing METTL3 and then relieved by upregulating MCAM (Fig. 6C). Flow cytometry analysis indicated that METTL3 deficiency facilitated the apoptosis of MG63 and U2OS cells, while MCAM elevation bated the effect (Fig. 6D and E). Transwell assay and wound-healing assay indicated that METTL3 deficiency led to the marked suppression in MG63 and U2OS cell invasion and migration, with MCAM enhancement ameliorated the effects (Fig. 6F and G). METTL3 deficiency decreased the protein levels of CyclinD1 and MMP9 in MG63 and U2OS cells, while these impacts were weakened by elevating MCAM (Fig. 6H and I). Taken together, METTL3 silencing impeded the malignant behaviors of OS cells by regulating MCAM.

### IGF2BP1 knockdown blocked the malignant behaviors of OS cells via MCAM

**Fig. 7:**
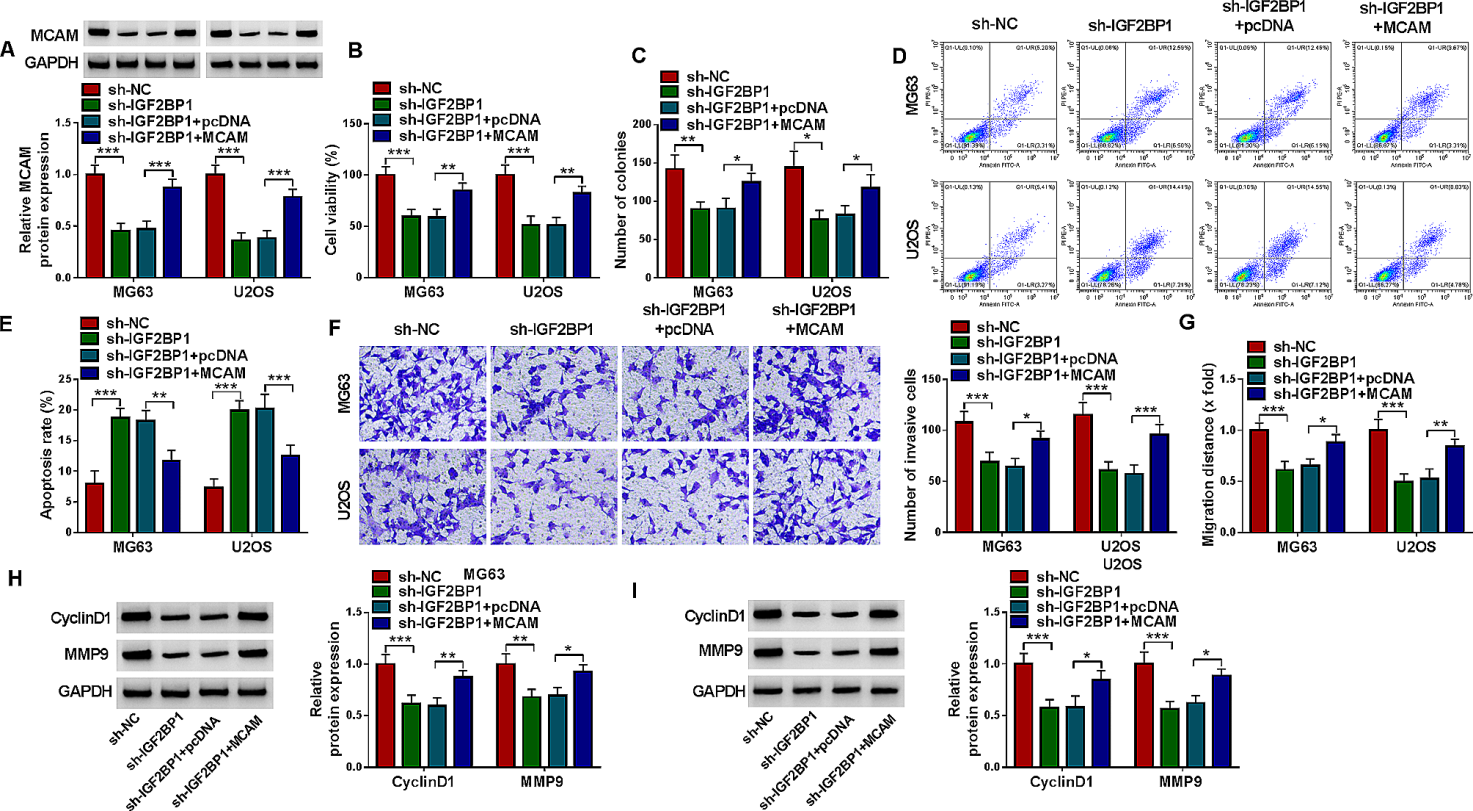
IGF2BP1 deficiency inhibited OS cell progression by modulating MCAM. MG63 and U2OS cells were transfected with sh-NC, sh-IGF2BP1, sh-IGF2BP1 + pcDNA or sh-IGF2BP1 + MCAM. (**A**) MCAM protein level was examined by western blot. (**B** and **C**) MG63 and U2OS cell viability and colony formation were assessed by MTT assay and colony formation assay, respectively. (**D** and **E**) The apoptosis of MG63 and U2OS cells was analyzed by flow cytometry analysis. (**F** and **G**) The invasion and migration of MG63 and U2OS cells were estimated by transwell assay and wound-healing assay. (**H** and **I**) The protein levels of CyclinD1 and MMP9 in MG63 and U2OS cells were measured by western blot. *P < 0.05, **P < 0.01, ***P < 0.001

The introduction of sh-IGF2BP1 markedly reduced MCAM protein expression in MG63 and U2OS cells, with MCAM overexpression vector transfection ameliorated the impact (Fig. 7A). MTT assay and colony formation assay indicated that IGF2BP1 knockdown repressed the viability and colony formation of MG63 and U2OS cells, while MCAM overexpression abated the effects (Fig. 7B and C). Flow cytometry analysis indicated that IFG2BP1 silencing-mediated promotion on MG63 and U2OS cell apoptosis was rescued by upregulating MCAM expression (Fig. 7D and E). The results of transwell assay and wound-healing assay indicated that IGF2BP1 deficiency apparently curbed the invasion and migration of MG63 and U2OS cells, whereas MCAM enhancement rescued the effects (Fig. 7F and G). Downregulation of IGF2BP1 decreased the protein levels of CyclinD1 and MMP9 in MG63 and U2OS cells, with MCAM overexpression abolished the impacts (Fig. 7H and I). All these findings suggested that IGF2BP1 silencing inhibited OS cell growth and metastasis and facilitated apoptosis through regulating MCAM expression.

### Transcription factor YY1 activated the transcriptional regulation of METTL3

**Fig. 8:**
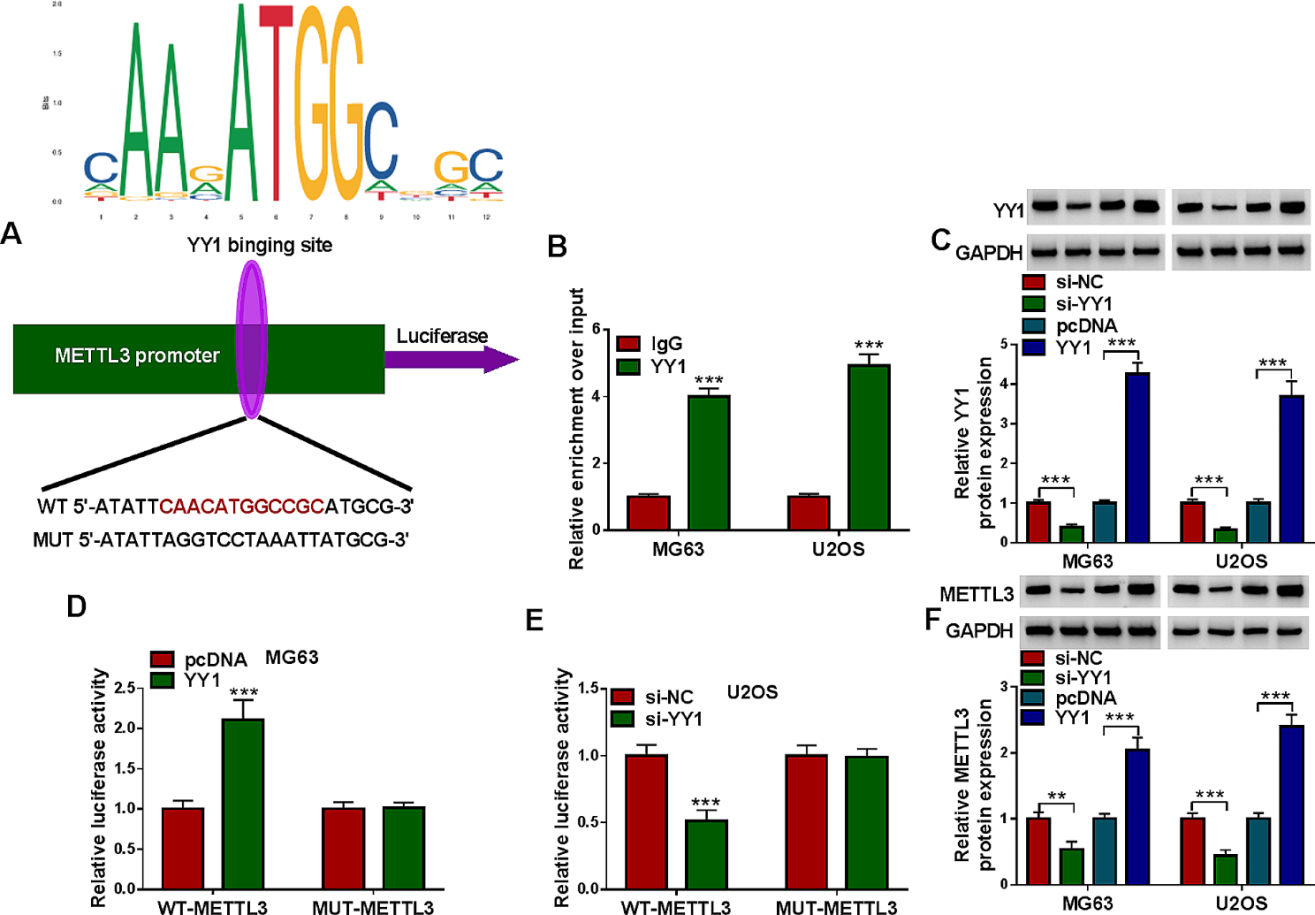
YY1 promoted the transcriptional regulation of METTL3 in OS cells. (**A**) Jaspar database predicted binding sites between transcription factor YY1 and METTL3 promoter. (**B**) ChIP assay was performed the combination between YY1 and METTL3. (**C**) The protein expression of YY1 in MG63 and U2OS cells transfected with si-NC, si-YY1, pcDNA or YY1 was determined by western blot. (**D** and **E**) The luciferase activity in MG63 with pcDNA/YY1 and WT-METTL3/MUT-METTL3 co-transfection and U2OS cells with si-NC/si-YY1 and WT-METTL3/MUT-METTL3 co-transfection was examined through dual-luciferase reporter assay. (**F**) METTL3 protein level in MG63 and U2OS cells transfected with si-NC, si-YY1, pcDNA or YY1 was measured via western blot. **P < 0.01, ***P < 0.001

As predicted by jaspar database, there were binding sites between transcription factor YY1 and METTL3 promoter (Fig. 8A). ChIP assay demonstrated the interaction between YY1 and METTL3 promoter (Fig. 8B). As presented in Fig. 8C, si-YY1 transfection decreased YY1 protein level and YY1 overexpression vector transfection increased YY1 protein level in MG63 and U2OS cells (Fig. 8C). Then dual-luciferase reporter assay showed the co-transfection of YY1 and WT-METTL3 markedly enhanced the luciferase activity in MG63 cells, and the co-transfection of si-YY1 and WT-METTL3 evidently repressed the luciferase activity in U2OS cells, while the luciferase activity of MUT-METTL3 luciferase reporter in both MG63 and U2OS cells was not affected (Fig. 8D and E). Furthermore, YY1 silencing decreased METTL3 protein expression and YY1 overexpression increased METTL3 protein expression in MG63 and U2OS cells (Fig. 8F). These findings indicated that YY1 facilitated METTL3 expression in OS cells.

### MCAM reversed the effect of METTL3 knockdown on tumor growth *in vivo*

**Fig. 9:**
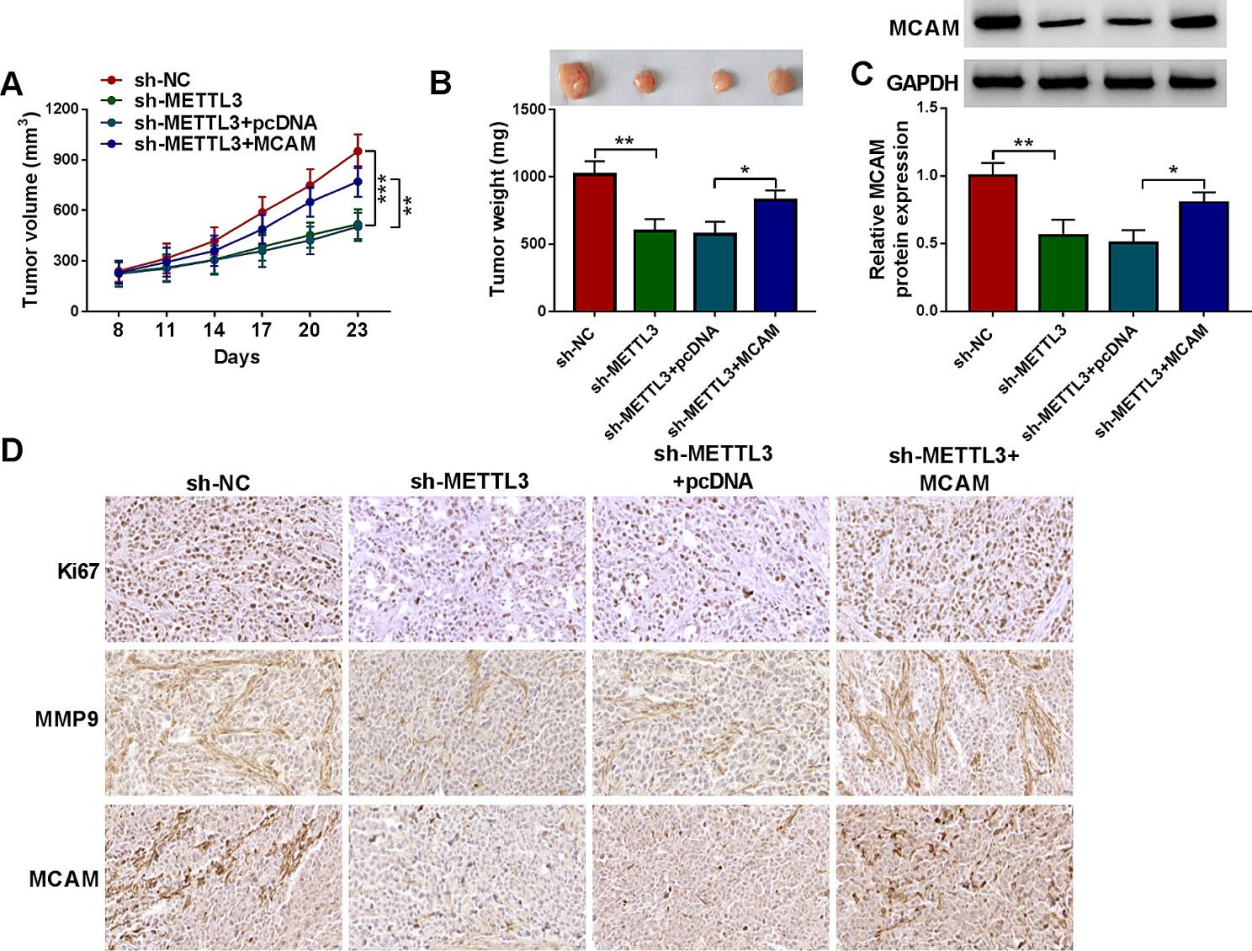
METTL3 knockdown blocked tumorigenesis by regulating MCAM. (**A**) Tumor volume was examined every 3 days. (**B**) Tumor weight in each group was measured. (**C**) MCAM expression in the collected xenograft tumors was examined by western blot. (**D**) Ki67, MMP9 and MCAM in the collected xenograft tumors was analyzed by IHC assay. *P < 0.05, **P < 0.01, ***P < 0.001

Finally, the role of MCAM in tumor growth was explored. Our results showed that METTL3 knockdown repressed xenograft tumor growth (including tumor volume and tumor weight) in vivo, while MCAM overexpression abated the effect (Fig. 9A and B). MCAM protein level in the tumors of sh-METTL3 group was reduced, but the effect was rescued in the tumors of sh-METTL3 + MCAM group (Fig. 9C). IHC assay showed that METTL3 knockdown suppressed the expression of Ki67, MMP9 and MCAM in the collected xenograft tumors, but MCAM overexpression abated the effect (Fig. 9D). These findings suggested that METTL3 silencing restrained tumor growth in vivo via MCAM.

## Discussion

Currently, the strategy for OS therapy is surgery combined with chemotherapy [26]. Multiple genes have been reported to serve as the prognostic biomarker in OS [27, 28]. Herein, we demonstrated the vital functions of MCAM, METTL3, IGF2BP1 and YY1 in OS.

MCAM presented an elevation in OS and related to overall survival of patients with OS and tumor cell metastasis [11, 29]. MCAM silencing inhibited the growth and migration and OS cells [10]. Moreover, MCAM (CD146) facilitated OS development via mediating tumorigenesis and angiogenesis [30]. In our present study, MCAM was verified to be upregulated in OS, which indicated the poor prognosis and was related to lymph node metastasis and advanced TNM stages. Furthermore, our findings proved that MCAM silencing restrained OS cell growth, invasion and migration and induced apoptosis, while MCAM overexpression exhibited the opposite results.

The occurrence and development of OS are closely related to epigenetic abnormal changes, and m6A is a common and extremely important epigenetic change [31]. Recently, the roles of m6A in human cancers attracted the researchers’ attention and it has been verified that the modification of m6A-related genes is associated with tumor progression [32, 33]. Through RM2target database and some experiments, MCAM contained the m6A methylation modification sites of METTL3 and METTL3 could regulate MCAM expression via m6A methylation modification. Zhou et al. declared that METTL3 was elevated in OS patients and aggravated OS cell growth and metastasis through enhancing the stability of DANCR mRNA via m6A modification [34]. Jiang et al. demonstrated that METTL3 facilitated OS malignancy via stabilizing HDAC5 mRNA in an m^6^A-dependent manner [35]. Miao et al. verified the oncogenic role of METTL3 in OS via modulating the m6A level of LEF1 [36]. Moreover, some previous articles showed that METTL3 altered the m6A methylation modification of MALAT1, DANCR, ZBTB7C and TRAF6 to facilitate OS cell growth and metastasis [13, 37–39]. However, the relation between METTL3 and MCAM was firstly investigated. Knockdown of METTL3 suppressed the growth and metastasis and promoted the apoptosis of OS cells, with MCAM upregulation abolished the effects. Besides, we found that METTL3 impeded tumor growth in vivo via regulating MCAM expression. Our outcomes suggested that METTL3 promoted the m6A level in MCAM to aggravated OS malignancy.

Furthermore, we firstly demonstrated that IGF2BP1 could regulate MCAM expression through m6A methylation modification. Moreover, IGF2BP1 mediated MCAM expression-affected by METTL3 in OS cells. Previous reports verified that IGF2BP1 was associated with OS cell growth and migration [40, 41]. Herein, IGF2BP1 was demonstrated to facilitate OS cell growth and metastasis by the m6A methylation modification of MCAM.

Additionally, transcription factor YY1 was demonstrated to activate the transcriptional regulation of METTL3. However, their relation in regulating OS progression was not investigated. Moreover, the exact role of YY1 in OS development still needs further exploration through in vitro and in vivo experiments.

In summary, we demonstrated that METTL3/IGF2BP1 promoted the malignancy of OS through the modification of MCAM. A novel regulatory axis of YY1-METTL3/IGF2BP1-MCAM was discovered in OS, which deepened the understanding of OS pathogenesis and might provide novel method for OS therapy.

### Electronic supplementary material

Below is the link to the electronic supplementary material.

Supplementary Material 1

## Data Availability

Data is available from the corresponding author upon reasonable request.
