# Supplementary figures and images for: METTL3 promotes the progression of osteosarcoma through the N6-methyladenosine modification of MCAM via IGF2BP1
Source: Biol Direct. 2024 Jun 7;19:44. doi: 10.1186/s13062-024-00486-x (PMC11157866; doi:10.1186/s13062-024-00486-x)

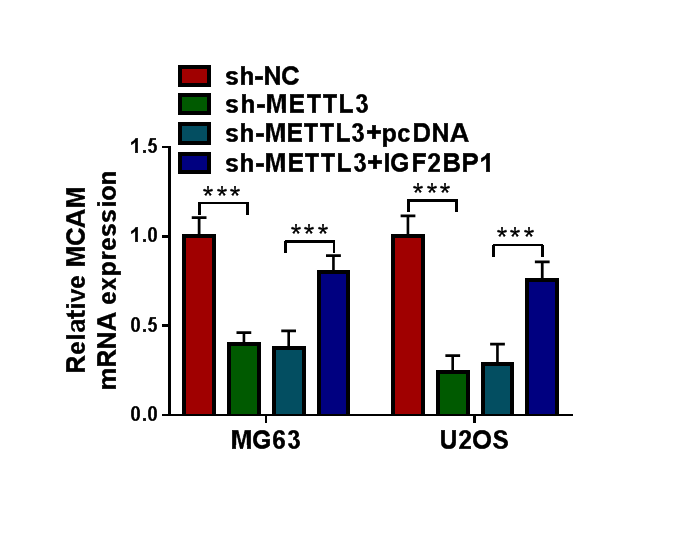

Supplement: Supplementary file 1 — Supplementary Material 1 [file 13062_2024_486_MOESM1_ESM.tif]
